# The shear rate promotes pinocytosis of extracellular dextran in platelets

**DOI:** 10.3233/CH-232075

**Published:** 2024-06-25

**Authors:** Masataka Inoue, Masahiro Ohwada, Nobuo Watanabe

**Affiliations:** a Functional Control Systems, Graduate School of Engineering and Science, Shibaura Institute of Technology, Saitama, Japan; b Department of Bio-Science and Engineering, Biofluid Science and Engineering Laboratory, College of Systems Engineering and Science, Shibaura Institute of Technology, Saitama, Japan

**Keywords:** Platelet, pinocytosis, dextran, shear rate, aggregation

## Abstract

**BACKGROUND:**

Several conventional studies focused on platelet pinocytosis for possible utilization as drug delivery systems. Although platelet pinocytosis is important in such utilization, the impact of the shear rate on pinocytosis is unclear.

**OBJECTIVE:**

Our objective was to investigate the relationship between shear rate and platelet pinocytosis *in vitro*. In addition, this study addressed the change in platelet aggregation reactivity with adenosine diphosphate (ADP) stimulation after pinocytosis.

**METHOD:**

Porcine platelet-rich plasma was mixed with fluorescein isothiocyanate (FITC)-conjugated dextran and incubated for 15 min under shear conditions of 0, 500, and 1500 s^-1^. After incubation, confocal microscopic scanning and three-dimensional rendering were performed to confirm the internalization of FITC-dextran into platelets. The amount of FITC-dextran accumulated via platelet pinocytosis was compared using flow cytometry at each shear rate. In addition, light transmission aggregometry by ADP stimulation was applied to platelets after pinocytosis.

**RESULTS:**

The amount of intracellular FITC-dextran increased with higher shear rates. Platelets with increased amounts of intracellular FITC-dextran did not show changes in the aggregation reactivity to ADP.

**CONCLUSIONS:**

A higher shear rate promotes platelet pinocytosis, but enhanced pinocytosis does not affect aggregation sensitivity, which is stimulated by ADP.

## 1 Introduction

Platelets are 2–4 μm in diameter and play an important role in hemostasis during their lifespan (5–12 days) [1–5]. Platelets also have been gaining attention as a delivery vehicle in drug delivery systems (DDS) for the treatment of cancer diseases [6–8]. In such previous studies, the anticancer drug called doxorubicin (DOX) was loaded into platelets. Related to the incorporation phenomenon of extracellular substances, receptor-mediated endocytosis and fluid-phase endocytosis are known as pathways [9], while the latter, receptor-independent uptake, is important for platelet utilization to DDS. Fluid-phase endocytosis is also called pinocytosis, the phenomenon in which small extracellular molecules that attach to the cell membrane is brought inside the cells. In a platelet study, canine platelets have shown that immunoglobulin uptake increased the protein level in the *α*-granules [10]. Through such fluid-phase endocytosis, it has been suggested that circulating platelets acquire immunoglobulins and albumin from blood plasma [11]. Furthermore, it is considered that the transport of proteins from plasma to *α*-granules continues throughout the lifespan of platelet with exposure tocirculation [12].

In proximal tubule cells and vascular endothelial cells, pinocytosis has been reported to be promoted by changes in membrane tension and calcium ion concentration due to shear rate stimulation [13, 14]. However, the effect of the shear rate on pinocytosis in platelets is not fully understood. From a rheological perspective, platelets are widely known to be exposed to a variety of shear rates in the blood circulation, ranging from 200 to 1600 s^–1^ [15]. When the shear rate exceeds the physiological range up to 4,000 s^–1^, the hydrodynamic stimulus causes platelet activation and aggregation [16–21]. Related to platelet activation and aggregation, they simultaneously release inside proteins or organic compounds like adenosine diphosphate (ADP). Despite this well-understood relationship between shear rate and release reactions from platelet, the relationship between shear rate and platelet pinocytosis has rarely been investigated.

The purpose of this study is to investigate the effect of the shear rate on the pinocytosis in porcine platelets using fluorescein isothiocyanate (FITC)-dextran as a fluid-phase marker during exposure to the shear rate through an *in vitro* experiment. Additionally, our objective is to determine changes in platelet aggregation sensitivity to ADP stimulation after pinocytosis. In the present study, we evaluated the level of FITC-dextran that platelets took up with confocal fluorescence microscopy and flow cytometry. In addition, light transmission aggregometry was performed on platelets after incorporation ofdextran.

## 2 Methods

### 2.1 Test blood samples

Porcine whole blood (*n* = 5) was collected at a slaughterhouse (Tokyo Shibaura Zoki K.K., Tokyo, Japan) and immediately anticoagulated with a 1:10 ratio of 3.24 wt% sodium citrate. To obtain platelet-rich plasma (PRP), 200 mL whole blood was centrifuged at 150×*g* for 15 min. Additionally, PRP was again centrifuged at 300×*g* for 10 min to reduce the amount of erythrocytes and leukocytes to the extent possible. Finally, the PRP platelet count was adjusted to around 300±50×10^9^/L with native plasma.

### 2.2 Shear loading a mixture of platelets and dextran

Before shear loading, 3.2 mL of PRP was mixed with 750 μL of FITC-conjugated dextran 70 s (100 μM, MW70,000, 90718; Sigma Aldrich, St. Louis, MO), a representative probe for fluid-phase endocytosis. The platelet mixture was incubated at 37°C with shear rates of 0 s^–1^, 500 s^–1^, and1,500 s^–1^ for 15 min using a custom-built blood shearing device. The detailed geometry of the shearing device has previously been described [22, 23]. The shear-loaded mixture was centrifuged at 2,000×*g* for 2 min. The supernatant containing FITC-dextran was replaced with 10 mL of phosphate buffered saline and the accumulated platelets at the bottom of the tube were gently dissolved to prevent platelet activation [24]. Such washing procedure was performed twice to remove the FITC-dextran. The platelet suspension was then divided for fluorescent labeling and light transmission aggregometry.

### 2.3 Fluorescent platelet staining and three-dimensional confocal microscopy

50 μL of sheared platelets were stained with 5 μL of Celltracker™ Orange CMRA dye (30 μM, C34551; Invitrogen, Waltham, MA) and incubated for 30 min at 37°C, according to the manufacturer’s protocol. To prevent deterioration in the cells and fluorophore, stained platelets were fixed with 2% paraformaldehyde within 4-hour time limit of platelet according to previous research [25] afterstaining.

Fluorescent labeled platelets were analyzed on a confocal microscope (FV1000; Olympus Corporation, Tokyo, Japan) using an oil-immersion×100 magnification objective lens (UPLFLN 100XO2 PH; Olympus Corporation) with a×3 optical zoom. The intensity emissions of FITC and Celltracker™ Orange CMRA were digitized through the internal sensor of 2,048×2,048 pixels, which corresponds to 40.94 μm×40.94 μm, in response to an excitation laser wavelength of 473 and 559 nm, respectively. The photon sensitivity for each emitted dye was set at 650 V and 530 V and the scanning speed of 2 μs for each pixel.

**Fig. 1:**
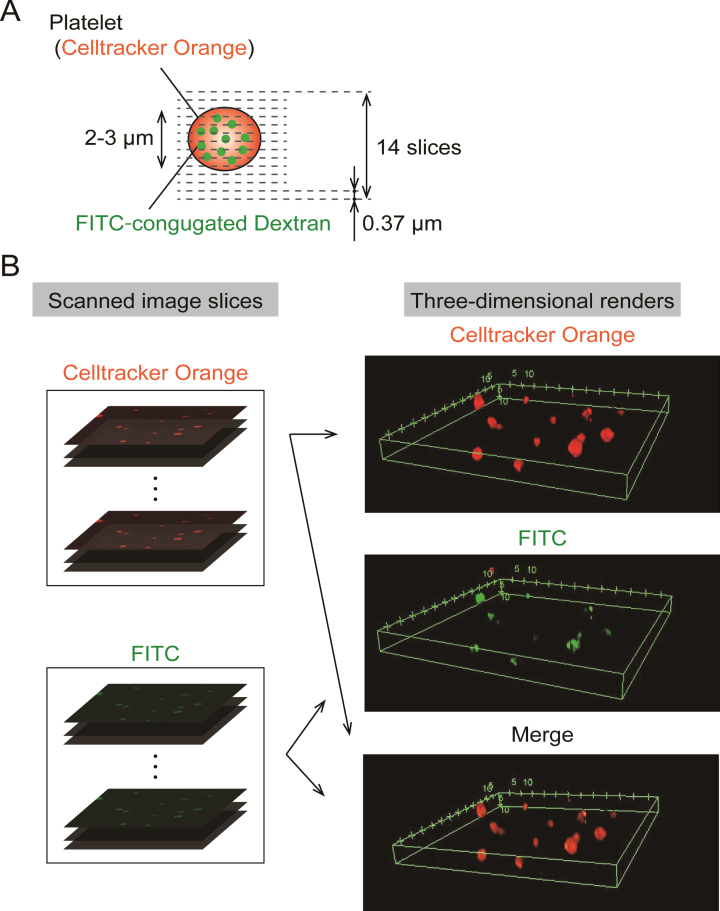
Three-dimensional renders were constructed with fluorescent images slice scanned by a confocal microscope. (A). Three-dimensional scanning set up: Series of scanned images was acquired by confocal microscopy with a×100 magnification objective lens at each depth of 0.37 μm and totally 14 slices were obtained. (B). Procedure for Construction of three-dimensional renders: Confocal scanned image series were rendered into three-dimensional images of FITC, Celltracker™ Orange CMRA and merge color.

A series of 14 slice images was obtained in the depth direction by moving the objective lens, and each step distance between slices was adjusted to 0.37 μm (Fig. 1A). The captured series of FITC, Celltracker™ Orange CMRA images were reconstructed to three-dimensional renderings using software provided by a microscope company (FV-10ASW, Olympus Corporation)(Fig. 1B).

### 2.4 Flowcytometric analysis of fluorescent platelets

The fluorescent intensity at the FITC wavelength was measured using a flow cytometer (BD FACS Area III™; Becton, Dickinson and Company, Franklin Lakes, NJ) to compare platelet pinocytosis of FITC-dextran at each shear rate. For the flowcytometric analysis, the stopping gate was set at 10,000 platelet cell events and the intensity of forward scatter versus FITC intensity was plotted. Additionally, as an analysis of FITC intensity, the fluorescent intensity for 10,000 platelets was averaged. The fold changes of the average fluorescent intensity at the shear rate of 500 s^-1^ and 1,500 s^-1^ were considered based on the average intensity without the shear rate as a baseline.

### 2.5 Evaluation of platelet aggregability using a light transmission meter

After shear loading, 180 μL of platelet suspension was diluted with native plasma to adjust the platelet number to around 300±50×10^9^/L. Coordinated plasma was analyzed using a light transmission meter (Hema Tracer 712; MC Medical Inc., Tokyo, Japan). In this analysis, aggregability was quantified to evaluate platelet sensitivity to agonist of aggregation after physiological amplitude of shear exposure with FITC-dextran. At 30 s before measurement, the platelet suspension was stimulated by the addition of 20 μL ADP (1 mM final concentration) and the level of light transmission was measuredfor 270 s.

### 2.6 Statistical analysis

Results were described as means±SEM (standard error of the mean). Normalized fluorescent intensity acquired by flow cytometry and light transmission level were analyzed with the Friedman test instead of the parametric one way repeated measures ANOVA. When a significant interaction was detected, a multiple comparison procedure with Dunnett’s test was performed based on the value without shear. A *P* value < 0.05 was considered statistically significant. All data were analyzed with commercially available software (MATLAB; MathWorks, Natick, MA).

## 3 Results

### 3.1 Three-dimensional confocal scanning of platelet pinocytosis for FITC-dextran

**Fig. 2:**
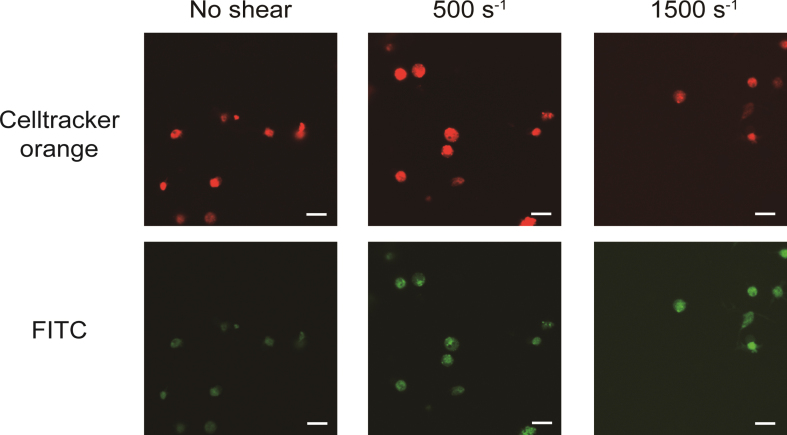
FITC-dextran was internalized into platelets as pinocytosis at all range of shear rates. Images were acquired by confocal microscope scanning for shear-loaded platelets incubated with FITC-dextran. The top three images are representative results for excitation of Celltracker^TM^ Orange CMRA dye (red in images) and the three bottom images are representative results for excitation of FITC (green in images). Scale bars, 10 μm.

**Fig. 3:**
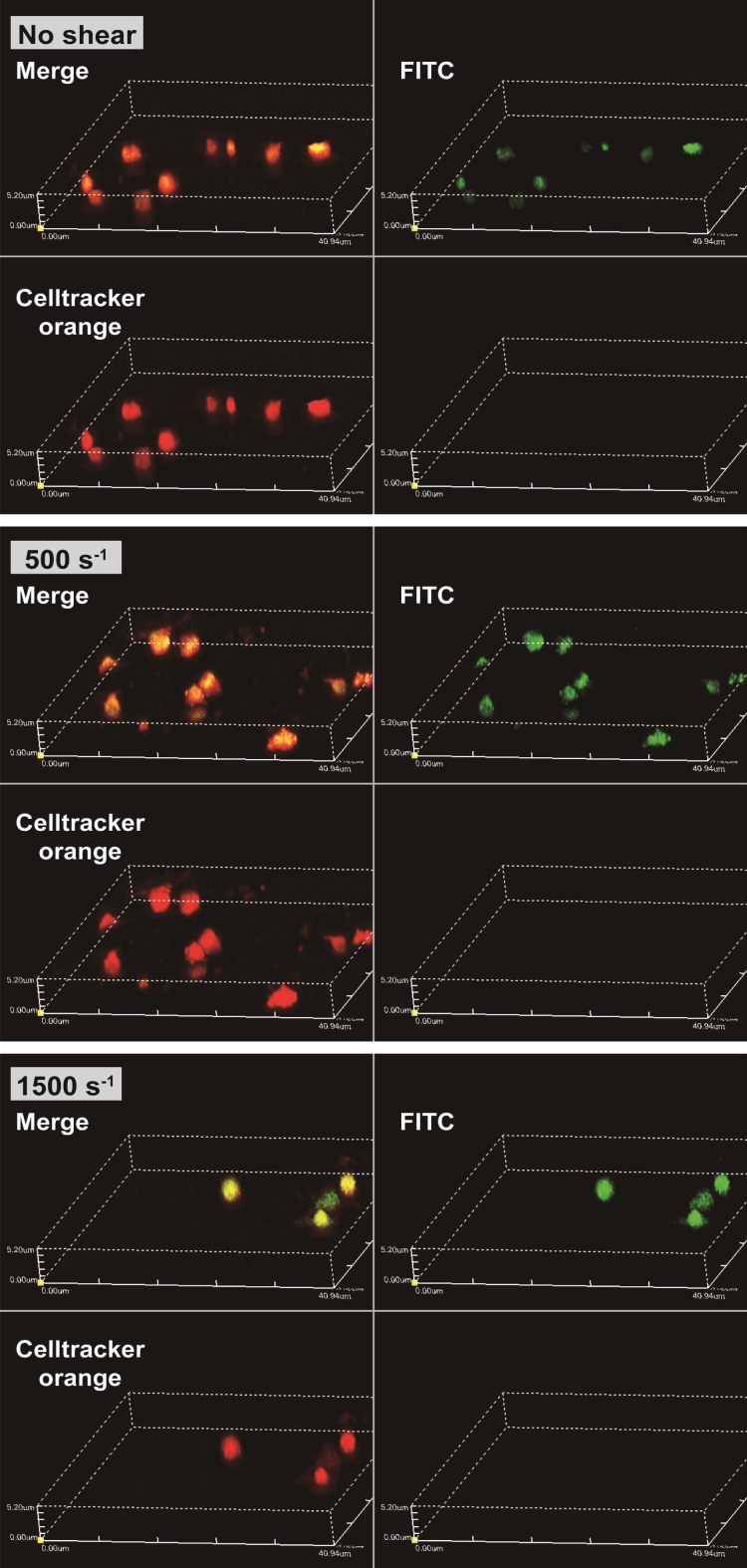
FITC-dextran that was incorporated into platelets as their pinocytosis and internalized in the spherical volume of platelets. Representative images of three-dimensional renders for all shear conditions and the 3D renders are shown for FITC, Celltracker™ Orange CMRA, and merge, intensities.

The obtained confocal images are shown in Fig. 2. The areas at the emission wavelength of Celltracker™ Orange CMRA dye are shown in red and FITC are shown in green. The diameters of red areas were around 2–3 μm, as the same range as platelets. At shear rates of 0 s^–1^, 500 s^–1^ and 1500 s^–1^, the emission of FITC areas (shown in green) were coincided with the area of Celltracker™ Orange CMRA dye. Three dimensional renderings of all shear rates at FITC, Celltracker™ Orange CMRA, and merge color are shown in Fig. 3. Excitation positions of FITC-dextran are internalized in the spherical fluorescent volume of Celltracker™ Orange CMRA for platelets.

### 3.2 FITC fluorescent intensity levels of platelets assessed by flow cytometry

**Fig. 4:**
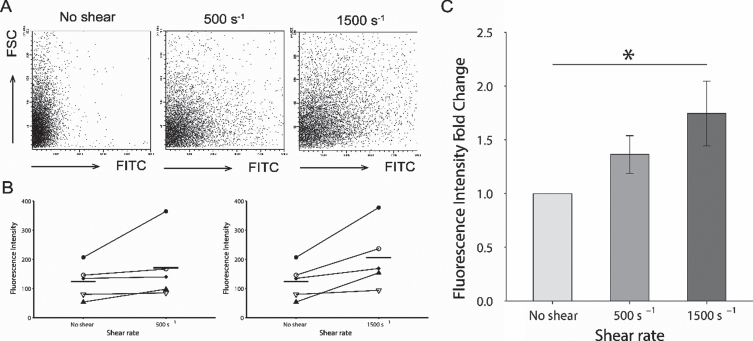
Comparison of fluorescent intensity acquired by flow cytometry. (A). Representative series of flow cytometric plots for each shear rate. The y-axis indicates the intensity of forward scatter for platelets and the x-axis indicates the intensity of FITC-dextran at shear rates of 0 s^–1^, 500 s^–1^, and 1500 s^–1^. (B). Averaged intensity levels of FITC under each shear conditions for each donor. Intensity levels are shown as the average for 10,000 platelets. Results are plotted for each donors and described as average±SEM, *n* = 5. (C). The fold change of the averaged fluorescent intensity of the FITC with comparison of shear rate. The fold changes in the fluorescent intensity at 500 s^–1^, and 1500 s^–1^ are based on the average intensity without shear condition as the baseline. Results are described as average±SEM, *n* = 5. Statistical analyses were performed using Dunnett’s test; ^*^P < 0.05.

The FITC intensity levels in platelets increased with the magnitude of the shear rate (Fig. 4A). The averaged FITC intensity levels of 10,000 platelets for each donor is shown in Fig. 4B. At the shear rate of 500 s^–1^ and 1,500 s^–1^ were increased from the ones at no shear condition. The fold change in the fluorescent intensity at 500 s^–1^ increased to 1.364±0.174 as shown in Fig. 3 C. For a shear rate of 1500 s^-1^, the fold change was shown as a significantly higher value of 1.746±0.301 compared to baseline (*P* < 0.05).

### 3.3 Light transmission aggregometry analysis of platelets after incubation with various shear rate conditions

**Fig. 5:**
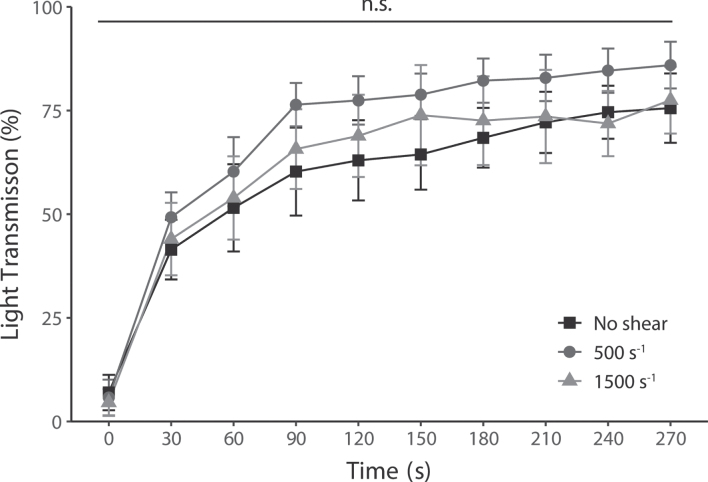
Platelet aggregation levels induced by ADP at all shear rates, nevertheless, had no change in their enhanced pinocytosis. Platelet aggregability was measured in each shear rate by light transmission. Increased light transmission shows platelet aggregation mediated by ADP-stimulated activation. Results are plotted for each test and average level±SEM, *n* = 5.

The time series of platelet light transmission levels after pinocytosis are shown in Fig. 5. Maximum light transmission levels were 75.6%, 85.9%, and 77.5 % without shear, 500 s^–1^, and 1500 s^–1^, respectively. Furthermore, there was no significant difference between the shear conditions at all elapsed times.

## 4 Discussion

Pinocytosis in platelets is one of the most important function as drug deliverer, however, the effect of the shear rate with physiological range on pinocytosis has not been clarified. In this study, FITC-dextran was used as a marker of porcine platelet pinocytosis and the relationship with the shear rate was investigated *in vitro*. We found that increased shear rate enhanced the platelets’ pinocytosis of FITC-dextran.

Platelet uptake of extracellular substances has been reported to involve both receptor-mediated endocytosis and pinocytosis [9]. Proteins such as fibrinogen are receptor-dependent, whereas FITC-dextran is taken up via the receptor-independent pinocytosis pathway [9]. Fluorescent three-dimensional scans in this study revealed that FITC-dextran was taken up by platelets without binding to the membrane surface. Previous studies related to platelet pinocytosis of dextran have considered that the lack of specific dextran receptors on the platelet membrane results in minimal adhesion to the membrane surface and immediate internal transfer of dextran [26]. Therefore, the results of the flow cytometric analysis in this study may primarily reflect the intensity of internally loaded FITC-dextran.

In addition, the open canalicular system (OCS) has also been proposed as a pathway for the uptake of dextran by platelets. Indeed, according to Behnke, small proteins and particles added to PRP *in vitro* appeared in the channels of the OCS without any change in platelet disc morphology, means without activation [26]. In terms of OCS in animals, platelets from some animal species, such as cow, camel, and horse, do not have OCS, while porcine platelets do [27]. Mentioning the size scales of OCS and dextran, previous studies have also shown that dextran with a MW of 80,000 and small latex spherules with a diameter of 87 nm was incorporated into OCS [26, 28]. Given that the FITC-dextran selected in this study has a lower MW of 70,000 than in previous studies and a smaller diameter of 11.6–14 nm [29, 30], it is reasonable that FITC-dextran was internalized into platelets. On the other hand, due to the limited resolution of confocal images, it was difficult to determine in detail the localization or uptake pathways of FITC-dextran in platelets.

This study evaluated the level of platelets pinocytosis of FITC-dextran under shear rate conditions of 0 s^-1^, 500 s^–1^ and 1500 s^–1^. The flow cytometric results indicated that the amount of internalized dextran increased at the higher shear rate. Exposed shear rates in our experiments correspond to the range in a shear stress of 0.6 Pa and 1.8 Pa (6 dyn/cm^2^ and 18 dyn/cm^2^), respectively, assuming a viscosity of 1.2 mPa·s for PRP. Shear rates below 3000 s^–1^ occur in our body, and previous studies have shown that platelets activate and aggregate at high shear loads beyond this biological range [16, 31]. In our shearing apparatus, proposed shear rate was lower than the threshold and likely prevented platelet activation. It suggests that the promotion of FITC-dextran internalization by an increased shear rate is independent of platelet activation.

The higher shear rate may have led to changes in cell membrane tension and consequently to increased endocytosis. In the past, a deep link between cell membrane tension and endocytosis has long been postulated [32]. Furthermore, studies related to proximal tubule cells consider the influx of intracellular calcium ions by fluid shear stimulation as the initiator of the cascade in pinocytosis [33]. Hence, in platelets, a similar trigger may have increased pinocytosis, since shear stimulation has been recognized to increase calcium ions [34, 35].

Although dextran internalization was enhanced, there was no change in platelet sensitivity to ADP stimulation and their aggregation. In previous studies, the addition of dextran *in vivo* and *in vitro* reduced platelet aggregation, which is interpreted as due to protein dilution in plasma, as represented by fibrinogen [36]. On the contrary, in this study, PRP after incubation with FITC-dextran was mixed with autologous plasma, so the proteins in the plasma were not diluted. Analogously to previous studies that dextran added to citrated PRP does not interfere with ADP-induced platelet aggregation [37, 38], we found that internalized dextran within platelets does not alter platelet aggregation function. As mentioned above, physiological shear rates not causing platelet activation were used in the present study, so it is easily interpreted that no change in the platelet aggregation response to ADP occurred. In summary, we hypothesize that the maximum shear rate of 1500 s^-1^ in our experiments was sufficient to promote pinocytosis by slightly increasing the intracellular calcium ion concentration, but not to lead to platelet activation. Furthermore, previous studies have shown that *in vivo* wall shear velocities range from 300 to 800 s^-1^ in the aorta and 500 to 1600 s^-1^ in the arterioles [39]. Thus, the applied shear rate in this study (500, 1500 s^-1^) gives us the insight to consider that plasma protein uptake by platelets in small arteries *in vivo* is greater compared to large arteries.

As a limitation of this study, to gain more insight into the mechanism of promotion of the pinocytosis by increased shear rate, more detailed evaluations should be required such as measuring changes in platelet calcium concentration and activation markers such as P-selectin on the platelet membrane. Furthermore, we know that platelet structure and activation pathways differ according to species [40], so porcine blood may not represent the platelet response in all animal species. It should be noted that pig platelets have less sensitivity to activate than human platelets [41], even though this study selected porcine platelets due to their many similarities to human platelets in that they have OCS and activation capabilities.

Consequently, the findings of this study can potentially contribute to the development of platelet-based drug delivery systems, in a more efficient method of internalization of drug internalization in platelet cells [6–8].

## 5 Conclusions

We successfully elucidated that platelet pinocytosis of FITC-dextran depended on the magnitude of shear rate. Furthermore, we clarified that their aggregation ability through ADP stimulation remained even after pinocytosis of FITC-dextran.
